# Striatal Activity Underlies Novelty-Based Choice in Humans

**DOI:** 10.1016/j.neuron.2008.04.027

**Published:** 2008-06-26

**Authors:** Bianca C. Wittmann, Nathaniel D. Daw, Ben Seymour, Raymond J. Dolan

**Affiliations:** 1 Wellcome Trust Centre for Neuroimaging, University College London, 12 Queen Square, London WC1N 3BG, UK; 2 Gatsby Computational Neuroscience Unit, University College London, 17 Queen Square, London WC1N 3AR, UK

**Keywords:** SYSNEURO

## Abstract

The desire to seek new and unfamiliar experiences is a fundamental behavioral tendency in humans and other species. In economic decision making, novelty seeking is often rational, insofar as uncertain options may prove valuable and advantageous in the long run. Here, we show that, even when the degree of perceptual familiarity of an option is unrelated to choice outcome, novelty nevertheless drives choice behavior. Using functional magnetic resonance imaging (fMRI), we show that this behavior is specifically associated with striatal activity, in a manner consistent with computational accounts of decision making under uncertainty. Furthermore, this activity predicts interindividual differences in susceptibility to novelty. These data indicate that the brain uses perceptual novelty to approximate choice uncertainty in decision making, which in certain contexts gives rise to a newly identified and quantifiable source of human irrationality.

## Introduction

Humans and other animals are naturally inquisitive and, in many circumstances, have a characteristic tendency to explore novel and unfamiliar stimuli and environments (Daffner et al., 1998; Ennaceur and Delacour, 1988; Hughes, 2007). Indeed, this tendency is exploited in marketing strategies whereby manufacturers of everyday consumable goods regularly remarket identical, or near-identical, products with novel packaging or advertising (Steenkamp and Gielens, 2003). Consumers' vulnerability to such manipulation may reflect the fact that, in naturalistic environments, novelty seeking can be strongly adaptive: because unfamiliarity normally tends to imply uncertainty, subsequent exploration carries with it the opportunity to discover unknown and potentially valuable outcomes.

Economic and computational models have formalized the adaptive value of information gathering, and, equally important, how this can be traded off against the substantial costs and risks entailed. A fully rational solution (in the sense of maximizing expected utility; e.g., Sanfey et al., 2006) quantifies the value of exploration in terms of uncertainty reduction and the beneficial effects of this knowledge on future choices (Gittins and Jones, 1974). In practice, this optimal approach is computationally laborious, and researchers in robotics and computer science have often employed a shortcut that encourages exploratory behavior in artificial agents by assigning a fictive “bonus” reward value to novel options (Brafman and Tennenholtz, 2003; Gittins and Jones, 1974; Kaelbling, 1993; Ng et al., 1999), that is, by treating novel stimuli as themselves rewarding. Here, we investigate the possibility that human brains employ a similar heuristic. Note that insofar as novelty does not perfectly signal an unknown option—as with the example of repackaged goods—this approach departs from that prescribed by a rational analysis, for instance by exploring unnecessarily.

The idea that novelty engages brain systems involved in appetitive reinforcement learning is supported by evidence that novel stimuli excite dopaminergic neurons in animals and also activate putatively dopaminergic areas in humans (Bunzeck and Duzel, 2006; Horvitz, 2000; Schultz, 1998). Computational theorists have interpreted these findings in terms of novelty bonuses (Kakade and Dayan, 2002). Furthermore, in a novelty paradigm modeled after classical conditioning procedures (Wittmann et al., 2007), a familiar cue trained to predict subsequent novelty itself activated midbrain, a response pattern that is reminiscent of dopaminergic responses to cues predicting reward as well as being characteristic of reinforcement learning (Dayan and Balleine, 2002; Schultz and Dickinson, 2000).

These data suggest that stimulus novelty might enhance exploratory choices in humans through engagement of circuits within a putative reward system, which encompasses midbrain, striatum, amygdala, orbitofrontal cortex, and mesial prefrontal cortex (cf. Knutson and Cooper, 2005). Nevertheless, despite this strong suggestive evidence, no functional links have been demonstrated between a biological “novelty bonus” signal and actual novelty-seeking behavior.

**Figure 1:**
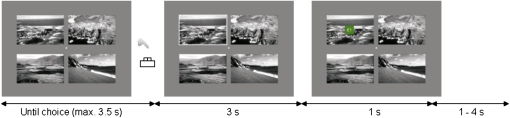
Experimental Design Following a familiarization phase, participants were shown four pictures on each trial and asked to choose one. Both familiarized and novel pictures were presented at randomized locations that changed on each trial. Each picture was repeated for an average of 20 trials and then replaced. Participants were informed that each picture had been assigned a unique probability of winning £1 that would not change as long as that picture was repeated. They were given feedback at the end of each trial indicating whether they had won or received nothing.

To investigate these links, we studied novelty-related decision making and associated brain activity in 15 healthy adults using functional magnetic resonance brain imaging (fMRI). We sought to test a computational hypothesis that brain systems associated with choice behavior, which are well described within reinforcement learning models, use novelty bonuses to encourage exploration of unfamiliar options. Participants performed a “four-armed bandit” choice task, in which the options were represented by four simultaneously presented landscape (“postcard”) images per trial (Figure 1). Each image, repeated over an average of 20 trials, was associated with a random, constant probability of winning money (one pound sterling). It was then replaced with another image with a new payoff probability. Subjects could only discover an option's reward probability by repeatedly sampling it, inducing a classic exploration/exploitation dilemma for subjects striving to maximize their earnings. Critically, we manipulated the novelty of images independently of reward value and uncertainty by familiarizing the subjects with half of the pictures (though not their associated reward probabilities) in a separate task before the scanning session. The images then used in the choice task were drawn pseudorandomly from the pre-exposed and novel sets; the associated payoff probabilities were also allocated pseudorandomly but with the same distribution for each set. This design ensures that novel options are no more uncertain, nor on average more valuable, than familiarized ones, allowing us specifically to examine a hypothesized mechanism of exploration directed toward perceptual novelty.

## Results

### Behavioral Novelty Preference

We fit participants' choices using a temporal-difference learning model (Sutton and Barto, 1998) similar to those used to account for choice behavior and neural signals in previous studies (Daw et al., 2006; Li et al., 2006; Tanaka et al., 2004). The model assumes that participants learn the value of each option and direct their choices toward those options predicted to be most valuable. In similar algorithms in artificial intelligence, novelty bonuses are often incorporated by “optimistically” initializing the starting value of new options to a higher level, encouraging exploration to determine their true value (Brafman and Tennenholtz, 2003; Ng et al., 1999).

To test for such bonuses, we included two parameters, representing the initial value attributed to novel and prefamiliarized pictures. The best-fitting parameters over subjects are shown in Table 1. We first tested whether this model accounted better for the subjects' choices than a simpler model that initialized both sets of pictures with the best shared initial value; it did (likelihood ratio test, 15 d.f., p < 0.005). Adopting the model with separate initial values, we found that novelty significantly enhanced exploration, as evidenced by the fact that, for the best-fitting parameters, the average expected value attributed to novel pictures (Q_n_) on their first introduction was significantly higher than the corresponding expected value for familiar pictures (Q_f_; mean Q_n_ = 0.41 ± 0.076 pounds over subjects; mean Q_f_ = 0.37 ± 0.071 pounds; paired t test p = 0.01). Put simply, this quantified the monetary value of novelty at approximately 4 pence.

Average reaction time (RT) for all choices was 1458 ± 80 ms. RTs did not differ between novel and familiar stimuli chosen on the trial when they were first introduced (1692 ± 110 ms and 1774 ± 126 ms, respectively). Scores on the novelty-seeking subscale of the TPQ ranged from 24% to 69% of the maximal score (mean = 52% ± SE 3.4). Individual participants' novelty bonuses, measured as a fraction relative to Q_f_, i.e., (Q_n_ - Q_f_)/Q_f_, ranged from −0.17 to 0.53 (mean = 0.12 ± SE 0.05).

### Striatal Reward and Novelty Signals

In terms of brain activity, we hypothesized that novelty bonuses would affect “prediction error” signals believed to influence learned value prediction and choice (Kakade and Dayan, 2002; McClure et al., 2003; O'Doherty et al., 2003; Pessiglione et al., 2006; Schultz, 1998; Tobler et al., 2006). To formally test this in our neuroimaging data (using SPM5), we ran two versions of the model to generate trial-by-trial prediction error signals for each subject. The first version was the novelty bonus model described above, while the second model applied the initial value of familiar stimuli to all stimuli, thus eliminating any impact of a novelty bonus. This second version was used to identify brain regions responding to a standard prediction error, and the difference between both prediction errors was then used to characterize areas in which neural activity was additionally correlated with a further error due to the novelty bonus. If neuronal prediction error activity is influenced by novelty bonuses, then it follows that it should correlate in the same brain area with both signals. We confined our analyses to ventral striatum and midbrain areas corresponding to our prior hypothesis (Kakade and Dayan, 2002).

**Figure 2:**
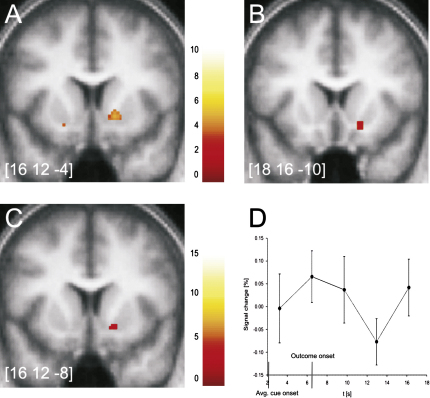
Ventral Striatal Response to Prediction Error and Novelty Peak coordinates are given in MNI space on all images. Color bars indicate T values. (A) Activation in right ventral striatum correlated significantly with reward prediction errors generated by the standard TD model (p < 0.001 uncorrected, p < 0.05 SVC, cluster > 5 voxels). (B) Activation in right ventral striatum correlated significantly with additional prediction error due to inclusion of a novelty bonus (p < 0.001 uncorrected, p < 0.05 SVC, cluster > 5 voxels). (C) Significant overlap between activation in right ventral striatum for the novelty bonus (see [B]) and activation obtained for standard model (see [A]) derived by inclusively masking (B) with (A) (p < 0.005, uncorrected, for both contrasts, cluster > 5 voxels). (D) Striatal activation time courses calculated for the first two trials a novel stimulus is chosen minus the first two choices of familiar stimuli, shown for the peak voxel correlating with the novelty bonus (MNI coordinates: 14, 20, −10). Trials are aligned by the time of reward outcome at 6.5 s; the average stimulus onset time is also indicated. Error bars indicate SEM.

Anticipation of reward has been shown to be associated with striatal activity in both active and passive tasks (Aron et al., 2004; Berns et al., 2001; Delgado, 2007; Knutson et al., 2000; Pagnoni et al., 2002; Samejima et al., 2005; Tanaka et al., 2004; Yacubian et al., 2007), a response that is correlated with prediction errors determined in temporal-difference learning models (Daw et al., 2006; McClure et al., 2003; O'Doherty et al., 2003; Rodriguez et al., 2006). Consistent with these findings, the standard prediction error generated by assuming identical initial expected values for novel and familiar stimuli correlated with activity in the ventral striatum (Figure 2A). Additionally, the component of prediction error due to the novelty bonus also correlated significantly with ventral striatal activity (Figure 2B). Figure 2C shows the significant overlap in the spatial expression of both activation maps. This finding is consistent with the computational model, which predicts that the full error signal is the sum of both components. Time courses from the peak voxel correlating with the bonus signal (Figure 2D) illustrate that the response to choice of novel (relative to familiarized) pictures has a biphasic shape. Note that a similar pattern is seen in the responses of dopaminergic neurons to novel stimuli in tasks not involving reward (Horvitz et al., 1997; Kakade and Dayan, 2002; Schultz, 1998) and is characteristic of the novelty bonus scheme in prediction error models, because more optimistic predictions lead to more negative prediction error when the actual reward is revealed.

### Measures of Individual Novelty Seeking

**Figure 3:**
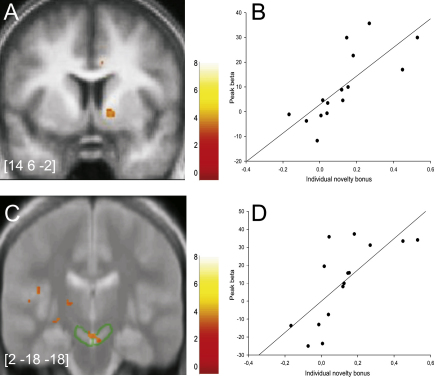
Individual Variation in Novelty Response Areas in which the level of activation by novelty bonus signal correlated significantly with individual subject measures of novelty seeking (p < 0.005 uncorrected, p < 0.05 SVC, cluster > 5 voxels). Peak coordinates are given in MNI space on all images. Color bars indicate T values. (A) Activation in right ventral striatum correlating with individual novelty seeking as measured in the behavioral task. Image is masked by “novelty bonus” contrast image from Figure 2B. (B) Peak beta values from (A) plotted against individual novelty-seeking measures. (C) Activation in right substantia nigra/ventral tegmental area correlating with individual novelty seeking as measured in the behavioral task, superimposed on a magnetization transfer image for better visualization of the substantia nigra (Bunzeck and Duzel, 2006). Image is masked by “novelty bonus” contrast image from Figure 2B. Substantia nigra is indicated by green circles. (D) Peak beta values from (C) plotted against individual novelty-seeking measures.

Finally, we reasoned that, if the identified striatal neural signals are indeed involved in novelty-seeking behavior, then we would also expect them to track interparticipant variability in this trait. Accordingly, we investigated whether the strength of the neural novelty bonus correlated with two behavioral measures of individual differences in novelty seeking. First, participants with higher novelty bonuses determined through model fits to their behavior in our task showed stronger novelty-bonus-related activation of the right ventral striatum and midbrain than did participants with lower behavioral novelty bonuses (Figure 3). By masking this analysis anatomically within the area activated on average by the novelty bonus in the group (Figure 2B), we verified that the striatal modulation of this activation indeed lies within the same region. Also, individual scores on the novelty-seeking subscale of Cloninger's Tridimensional Personality Questionnaire (TPQ) correlated with the degree of novelty-bonus activity in the left ventral striatum (see Figure S1 available online). These modulations (and the midbrain correlation with novelty seeking from the choice fits) lie outside the mask generated from the novelty-bonus group average but within areas that are known to be involved in reward and novelty processing (Wittmann et al., 2007, 2005). As predicted, there was no correlation of striatal or midbrain novelty signals with the harm-avoidance and reward-dependence subscales of the TPQ and no correlation of activations for the base component of the prediction error with any of the novelty-seeking measures.

## Discussion

Our data show that novelty enhances behavioral exploration in humans in the context of an appetitive reinforcement learning task. Participants' actual choices were best captured in a model that introduced higher initial values for novel stimuli than for prefamiliarized stimuli. This computationally defined novelty bonus was associated with activation of ventral striatum, suggesting that exploration of novelty shares properties with reward processing. Specifically, the observed overlap of novelty-related and reward-related neural components of prediction error signals supports this interpretation. The observation that activation by novelty bonuses in both striatal and midbrain areas correlated with individual novelty-seeking scores points to a functional contribution of the mesolimbic system to novelty-related enhancement of choice behavior.

All of these findings are consistent with a specific computational and neural mechanism (Kakade and Dayan, 2002), namely that a dopaminergic prediction error signal for reinforcement learning reports a novelty bonus encouraging exploration. Such a model had been originally advanced to explain dopaminergic neuron responses to novel stimuli in passive, nondecision tasks (Horvitz et al., 1997; Schultz, 1998), a response pattern that has also been suggested in humans (Bunzeck and Duzel, 2006; Wittmann et al., 2007). By linking a bonus-related neural signal to actual novelty-seeking behavior, the present study provides evidence to support a model of dopamine-driven novelty exploration. While it is not possible to identify definitively the neural source underlying fMRI signals, recent results support an inference that striatal prediction error signals have a dopaminergic basis, because they are modulated by dopaminergic drugs (Pessiglione et al., 2006; Yacubian et al., 2006). Also, given that fMRI does not allow inference of causality from correlations of brain activity with behavior, alternative explanations for our findings are possible. For instance, areas outside of the mesolimbic system could mediate the exploration effect of novelty, and the striatal activations might then reflect these choices. However, in directly contrasting exploratory to exploitative choices (as in Daw et al., 2006), we did not find novelty- or exploration-related activity in frontopolar cortex, a candidate region outside the midbrain (Daw et al., 2006).

Computational models stress the necessity to overcome exploitative tendencies in order to optimize decision making under uncertainty (Gittins and Jones, 1974). One solution to this is the introduction of an exploration bonus to guide decisions toward uncertain options (Gittins and Jones, 1974; Kaelbling, 1993). Here, we provide evidence for a specific version of such a bonus that uses novelty as a signal for uncertainty (Brafman and Tennenholtz, 2003; Kakade and Dayan, 2002; Ng et al., 1999). Notably, a bonus directed toward uncertainty per se was not evident, either neurally or behaviorally, in a previous study of gambling involving an n-armed bandit task, in which uncertainty arose due to a gradual change in the unknown payoffs but without accompanying perceptual novelty (Daw et al., 2006). The differences between the tasks may explain why, in the previous study but not the present one, exploratory choices were found to be accompanied by BOLD activity in frontopolar cortex, a region broadly associated with cognitive control. Psychologically, exploration in a familiar context, as in the earlier study, requires overriding not only a tendency to exploit known highly rewarding stimuli but also a tendency to avoid previously low-valued stimuli. However, novel options, like those used here, may not only be attractive due to a novelty bonus, but crucially have no history of negative feedback, perhaps reducing the demand for cognitive control to encourage their exploration.

Computationally, the present findings point to the likelihood that humans use perceptual novelty as a substitute for true choice uncertainty in directing exploration. This would explain why they had a greater tendency to explore perceptually novel options even when no more uncertain and also why our previous study (Daw et al., 2006) did not detect exploration directed toward uncertainty without perceptual novelty. Such a scheme is common in artificial intelligence (Brafman and Tennenholtz, 2003; Ng et al., 1999), because it is so easily implemented by optimistic initialization. Additionally, it seems to be a plausible neural shortcut, because novelty is likely to be a reliable signal for uncertainty in the natural world. Physiologically, this appears to be implemented by using the same system to process the motivational aspects of standard reward.

To be sure, on a rational analysis, the degree to which exploration is net beneficial depends on a number of circumstantial factors, including for instance how dangerous unexplored alternatives are likely to be. Computationally, this points to an important requirement that the degree of novelty seeking needs to be carefully tuned to appropriate levels (there are some proposals for the neural substrates for similar “metalearning” processes; Doya, 2002). Behaviorally, this point resonates with the fact that animals' novelty preferences exhibit a great deal of subtle contextual sensitivity (Hughes, 2007). Rats, for instance, avoid novel foods (presumably due to serious risk of illness), and fear-promoting stimuli such as electric shocks can also promote novelty avoidance on some tasks. Such phenomena are not inconsistent with our account of novelty seeking in the present (safe) context; indeed, we would infer that our approach could easily be extended to quantify the effects of factors such as fear.

Finally, while the novelty bonus may be a useful and computationally efficient heuristic in naturalistic environments, it clearly has a downside. In humans, increased novelty seeking is associated with gambling and addiction (Hiroi and Agatsuma, 2005; Spinella, 2003), disorders that are also closely linked to dopaminergic pathophysiology (Chau et al., 2004; Reuter et al., 2005). More generally, the substitution of perceptual novelty for choice uncertainty represents a distinct, albeit slight, departure from rational choice that, as in our task, introduces the danger of being sold old wine in a new skin.

## Experimental Procedures

### Participants

Twenty healthy adults participated in the experiment, four of which had to be excluded for technical problems with stimulus presentation and scanner sequence software and another for electing to leave the experiment before it was complete. Fifteen right-handed participants (mean age, 26.1 ± 1.2; seven male) remained in the analysis. All participants gave written informed consent to participate, and the study was in accordance with the guidelines of the local ethics committee.

### Behavioral Paradigm

#### Familiarization Procedure

Prior to scanning, participants underwent two familiarization sessions that included 32 pictures from a set of 64 grayscale landscape photographs with normalized luminance and contrast. Each picture was presented four times per session in randomized order. In the first session, participants were asked to look at the pictures attentively without responding, while in the second session they were asked to respond to each picture per button press, indicating whether there was a building in the picture.

#### Prescanning

Participants received written instructions on the decision-making task, including the information that they would receive 20% of their winnings at the end of the experiment. They also completed a short button response training to ensure that their responses reflected their choices and a short training version of the task (Figure 1).

#### Scanning Task

Participants engaged in three sessions of 17.5 min length, each containing 100 trials of 8.5–11.5 s duration. On each trial, participants were presented with four pictures (visible on a screen reflected in a head coil mirror) and selected one depending on its location on the screen (top left, top right, bottom left, bottom right), using a button box with their right hand. If they did not choose a picture within 3.5 s, the feedback “No response” was presented on the screen for 6.5 s to signal an invalid trial. On valid trials, a frame was shown around the chosen picture, and feedback (£1 on a green square background or £0 on a blue square background) was presented 3 s later, superimposed on the chosen picture. A variable fixation phase (1–4 s) followed. Participants received either £1 or nothing, depending on the reward probability associated with the chosen picture. Each picture had been assigned a random reward probability (mean value: 0.33) that was not changed in the course of the experiment.

Each picture was repeated for an average of 20 trials (range: 5–35). The location of pictures was changed randomly on each trial, so that a decision could not be based on habitual responding with the same finger. In 20% of trials, one of the pictures was exchanged for another picture that had either been familiarized or was novel (=30 switches to either category in total).

After scanning, participants completed Cloninger's Tridimensional Personality Questionnaire (Cloninger et al., 1991), which tests for personality differences in three dimensions defined as novelty seeking, reward dependence, and harm avoidance.

### Behavioral Analysis

We characterized each subject's trial-to-trial choices using a temporal-difference learning model with four free parameters. The model assumes that the probability of choosing picture *c* (out of the four available options) on trial *t* is P(c(t)=c,t)∝exp(β⋅Q(c,t)); that is, softmax in Q(c,t), the presumed value of the option on that trial. The inverse temperature parameter *β* controls the exclusivity with which choices are directed toward higher-valued options.

According to the model, the values *Q* were learned from experience using a standard delta rule, Q(c(t),t+1)=Q(c(t),t)+ν⋅δ(t). Here, the value of the chosen option is updated according to the error signal δ(t)=r(t)−Q(c(t),t), which measures the mismatch between the reward delivered, r(t) (i.e., 1 or 0) and the value expected. *ν* is a learning rate parameter (values for options not chosen were not changed).

The initial values of each picture, Q(c,0), are set to $Q_{f}$ (a free parameter) if the picture had been pre-exposed during the familiarization phase, and to parameter $Q_{n}$ if not. The difference $Q_{n}-Q_{f}$ therefore confers differential initial value for non-pre-exposed pictures when first presented; if this difference is positive (a “novelty bonus”; Kakade and Dayan, 2002; Ng et al., 1999), it favors the choice of novel items when they first become available.

We optimized the parameters for each subject individually to maximize the likelihood of his or her observed sequence of choices, $\prod_{t}P\left(c\left(t\right),t\right)$, where the underlying values Q(c,t) were computed using the model and the preceding sequence of actual observed choices c(1…t−1) and rewards r(1…t−1). We also, separately, fit a nested model in with the initial values constrained to be equal, i.e., $Q_{n}=Q_{f}$, and compared the two models on the entire data set (pooled over all subjects) using a likelihood ratio test.

The best-fitting estimates for each parameter were then treated as a random variable instantiated for each subject (equivalently, we treated all parameters as random effects and estimated the moments of the group distribution using the summary statistics procedure [Holmes and Friston, 1998]). Because of a degeneracy in the model in some regimes (specifically, when *ν* is very small and consequently the *Q*s are consistently very far from asymptote), it was not possible to obtain reliable parameter estimates for one subject, who was therefore excluded from the estimates of the average parameters. Because the degeneracy manifests through poorly constrained but at the optimum aberrantly large and small (respectively) values of *β* and *ν*, the exclusion or inclusion of this subject had no appreciable effect on the reported hypothesis tests involving $Q_{f}$ and $Q_{n}$, or on the model comparison.

To generate model-based regressors for the imaging analysis, the learning model was simulated using each subject's actual sequence of rewards and choices to produce per-subject, per-trial estimates of the values Q(c,t) and error signals δ(t). The free parameters were taken to be top-level mean estimates from the random-effects model (i.e., the mean of the individual parameter estimates; this regularizes the individual estimates, which we have previously found to be noisy for these purposes, e.g., Daw et al., 2006).

To study the effects of the novelty bonus on the prediction error, we repeated the simulations, but taking $Q_{n}=Q_{f}$—that is, eliminating any bonus for non-pre-exposed pictures. This generated a second sequence of values $Q_{base}\left(c,t\right)$ and prediction errors $\delta_{base}\left(t\right)$, reflecting baseline values without the additional effects of the novelty bonus. For the purpose of regression, we decomposed the values Q(c,t) and δ(t) into the sums $Q_{base}\left(c,t\right)+Q_{add}\left(c,t\right)$ and $\delta_{base}\left(t\right)+\delta_{add}\left(t\right)$ of the baseline values plus additional increments for the effects of the bonus. We computed $Q_{add}\left(t\right)=Q\left(t\right)-Q_{base}\left(t\right)$ and $\delta_{add}\left(t\right)=\delta \left(t\right)-\delta_{base}\left(t\right)$. Together with a standard general linear analysis, this additive decomposition allowed us to study the contribution of baseline and bonus-related components of the prediction error signal to BOLD activity and to test the hypothesis that both components summate to produce the full error signal. Note that the bonus has a characteristic pattern of effects on the values and errors, which is not wholly confined (for instance) only to trials when a novel picture is first offered. For instance, if $Q_{n}>Q_{f}$ then $Q_{add}>0\text{and}\delta_{add}<0$ whenever a nonfamiliarized option is chosen.

### fMRI Procedures

The functional imaging was conducted using a 1.5 Tesla Siemens Sonata MRI scanner to acquire gradient echo T2^∗^-weighted echo-planar images (EPI) with blood oxygenation level dependent (BOLD) contrast. We employed a special sequence designed to optimize functional sensitivity in OFC and medial temporal lobes. This consisted of tilted acquisition in an oblique orientation at 30^∗^ to the AC-PC line, as well as application of a preparation pulse with a duration of 1 ms and amplitude of −2 mT/m in the slice selection direction. The sequence enabled 36 axial slices of 3 mm thickness and 3 mm in-plane resolution to be acquired with a repetition time (TR) of 3.24 s. Coverage was obtained from the base of the orbitofrontal cortex and medial temporal lobes to the superior border of the dorsal anterior cingulate cortex. A field map using a double echo FLASH sequence (64 oblique transverse slices, slice thickness = 2 mm, gap between slices = 1 mm, TR = 1170 ms, α = 90°, short TE = 10 ms, long TE = 14.76 ms, BW = 260 Hz/pixel, PE direction anterior-posterior, FOV = 192 × 192 mm^2^, matrix size 64 × 64, flow compensation) was recorded for distortion correction of the acquired EPI images. Participants were placed in a light head restraint within the scanner to limit head movement during acquisition. Functional imaging data were acquired in three separate 332 volume runs. A T1-weighted structural image, local field maps, and an inversion recovery EPI (IR-EPI) were also acquired for each subject. Scanning parameters were the same as for the EPI sequence but with full brain coverage.

### fMRI Analysis

Preprocessing and data analysis were performed using Statistical Parametric Mapping software implemented in Matlab (SPM5; Wellcome Department of Imaging Neuroscience, Institute of Neurology, London, UK). Using the FieldMap toolbox (Hutton et al., 2002, 2004), field maps were estimated from the phase difference between the images acquired at the short and long TE. The EPI images were corrected for distortions based on the field map (Hutton et al., 2002) and the interaction of motion and distortion using the Unwarp toolbox (Andersson et al., 2001; Hutton et al., 2004). EPI images were then spatially normalized to the Montreal Neurological Institute template by warping the subject's anatomical IR-EPI to the SPM template and applying these parameters to the functional images, transforming them into 2 × 2 × 2 mm sized voxels, smoothed using an 8 mm Gaussian kernel.

For statistical analysis, the data were scaled voxel-by-voxel onto their global mean and high-pass filtered. Each trial was modeled with impulse regressors at two time points: the time of the presentation of the pictures, which was taken to be the time of the decision, and the time of presentation of the outcome (3 s after key press). These events were modulated by parametric regressors simulating the baseline prediction error signal and the additional component to the error due to the novelty bonus. The baseline prediction error was defined as $Q_{base}\left(c\left(t\right),t\right)$ at the time the pictures were presented on trial *t* (Morris et al., 2006) and as $\delta_{base}\left(t\right)$ at the time the outcome was revealed. The novelty bonus contribution was modeled as $Q_{add}\left(c\left(t\right),t\right)$ and $\delta_{add}\left(t\right)$ at the same time points.

These regressors were then convolved with the canonical hemodynamic response function and its temporal derivative (Friston et al., 1998) and entered as separate orthogonalized regressors into one regression analysis against each subject's fMRI data using SPM, allowing independent assessment of the activations correlating with each model's predictions. The six scan-to-scan motion parameters produced during realignment were included as additional regressors in the SPM analysis to account for residual effects of scan-to-scan motion. To enable inference at the group level, the coefficient estimates for the two model-based regressors from each individual subject were taken to allow second-level, random-effects group statistics to be computed. To investigate how individual variation in novelty seeking impacted bonus-related BOLD activity, we included the normalized per-subject novelty bonus (Q_n_ − Q_f_)/Q_f_ (computed using the individual estimates of these parameters from the behavioral analysis) as a second-level regressor.

Results are reported in areas of interest at p < 0.001 uncorrected. The predicted activations in the ventral striatum were further tested using a spherical small-volume correction (SVC) centered on the peak voxel, with a radius of 9 mm, corresponding to the 3.43 cm^3^ volume of the putamen (Anastasi et al., 2006). All behavioral averages are given as mean values ± SE. To better localize midbrain activity, the relevant activation maps were superimposed on a mean image of 33 spatially normalized magnetization transfer (MT) images acquired previously (Bunzeck and Duzel, 2006). On MT images, the substantia nigra can be easily distinguished from surrounding structures (Eckert et al., 2004).

To illustrate time courses, we conducted an additional regression analysis on the voxel of peak activation for the bonus regressor using a flexible basis set of 1 TR duration finite impulse responses. Impulses were aligned according to the time of outcome reveal. Four trial types were modeled separately: the first two choices of a novel image, the first two trials of a familiar image, and (as effects of no interest) the remaining trials divided into two groups according to win versus loss.

## Figures and Tables

**Table 1:** Parameter Estimates for the Behavioral Model, Shown as Mean (Over Subjects) ± 1 SE

Learning rate ν	0.23 ± 0.038
Softmax inv. temperature β	8.5 ± 1.2
Initial value, familiarized *Q_f_*	0.37 ± 0.071
Initial value, novel *Q_n_*	0.41 ± 0.076

Due to poor identification of β and ν, one subject is omitted from these averages.
