# Supplementary material for: Striatal Activity Underlies Novelty-Based Choice in Humans
Source: Neuron. 2008 Jun 26;58(6):967–73. doi: 10.1016/j.neuron.2008.04.027 (PMC2535823; doi:10.1016/j.neuron.2008.04.027)

Neuron, Volume *58*

Supplemental Data

Striatal Activity Underlies Novelty-Based Choice in Humans

Bianca C. Wittmann, Nathaniel D. Daw, Ben Seymour,
and Raymond J. Dolan

Table S1.

Prediction error response: Anatomical locations of regions correlating with the standard TD model. Data are thresholded at p<0.001, uncorrected, and only clusters with >5 voxels are reported.

**Area**	**Left / Right**	**MNI coordinates**			**T value**
		**x**	**y**	**z**	
Insula	L	-28	22	8	4.09
Ventral putamen	R	16	12	-4	5.25
	L	-20	14	-10	4.25
Thalamus	L	-12	-14	6	4.63
	L	-20	-22	-2	4.47
	R	18	-26	-2	4.38
Parahippocampal gyrus, BA 19	L	-20	-74	-4	7.1
Middle occipital gyrus, BA 19	L	-32	-82	12	4.12
Middle occipital gyrus, BA 18	R	22	-86	-14	10.94

Table S2.

Novelty bonus response: Anatomical locations of regions with activity correlating with additional prediction error due to inclusion of a novelty bonus. Data are thresholded at p<0.001, uncorrected, and only clusters with >5 voxels are reported.

**Area**	**Left / Right**	**MNI coordinates**			**T value**
		**x**	**y**	**z**	
Middle frontal gyrus, BA 46	R	46	34	18	**4.41**
Inferior frontal gyrus, BA 47	L	-14	30	-18	**5.54**
	R	32	26	2	**4.11**
Nucleus accumbens	R	14	20	-10	**4.63**
Insula, BA 13	R	36	26	20	**5.48**
	R	38	20	8	**4.72**
	R	40	4	22	**4.27**
	R	50	-22	24	**4.3**
	L	-38	-40	22	**5.41**
Superior frontal gyrus, BA 8	L	-6	12	54	**5.29**
Inferior frontal gyrus, BA 9	R	46	6	32	**4.36**
Medial frontal gyrus, BA 6	R	8	-2	54	**4.5**
Precentral gyrus, BA 6	L	-38	0	26	**5.95**
Cingulate gyrus, BA 32	R	12	8	44	**6.13**
Inferior parietal lobule, BA 40	L	-58	-22	26	**4.02**
Postcentral gyrus, BA 3	L	-32	-24	50	**4.29**
Postcentral gyrus, BA 2	R	40	-28	42	**5.71**
Inferior parietal lobule, BA 40	L	-42	-34	44	**4.62**
Posterior cingulate, BA 31	L	-20	-60	20	**6.66**
Middle occipital gyrus, BA 19	L	-30	-68	-12	**15.72**
	R	44	-74	2	**4.66**
	L	-42	-80	14	**4.15**
Thalamus	R	8	-12	4	**5.47**
	L	-18	-20	4	**5.32**
	L	-6	-26	-6	**5.27**

Figure S1.

Activation in left ventral striatum correlating with individual novelty seeking as measured by the TPQ.

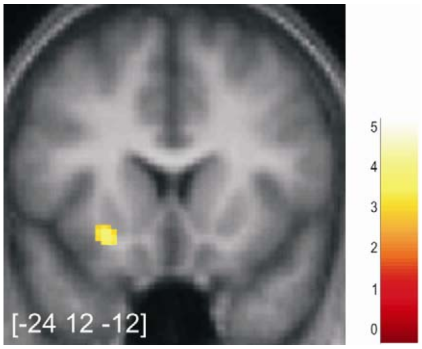

Supplement: Document S1. Supplemental Tables and Figure [file mmc1.pdf]
